# Impact of acute diabetes decompensation on outcomes of diabetic patients admitted with ST-elevation myocardial infarction

**DOI:** 10.1186/s13098-018-0357-y

**Published:** 2018-07-17

**Authors:** Mayada Issa, Fahad Alqahtani, Chalak Berzingi, Mohammad Al-Hajji, Tatiana Busu, Mohamad Alkhouli

**Affiliations:** 1 Department of Medicine, West Virginia University, Morgantown, WV USA; 2 Division of Cardiology, West Virginia University, Morgantown, WV USA; 3 West Virginia University Heart & Vascular Institute, 1 Medical Drive, Morgantown, WV 26505 USA

**Keywords:** Myocardial infarction, Diabetic ketoacidosis, Hyperosmolar hyperglycemic state, Coronary angiography, Coronary stenting

## Abstract

**Background:**

Acute hyperglycemia is associated with worse outcomes in diabetic patients admitted with ST-elevation myocardial infarction (STEMI). However, the impact of full-scale decompensated diabetes on STEMI outcomes has not been investigated.

**Methods:**

We utilized the national inpatient sample (2003–2014) to identify adult diabetic patients admitted with STEMI. We defined decompensated diabetes as the presence of diabetic ketoacidosis (DKA) or hyperglycemic hyperosmolar state (HHS). We compared in-hospital morbidity and mortality and cost between patients with and without diabetes decompensation before and after propensity-score matching.

**Results:**

A total of 73,722 diabetic patients admitted with STEMI were included in the study. Of those, 1131 (1.5%) suffered DKA or HSS during the hospitalization. After propensity-score matching, DKA/HHS remained associated with a significant 32% increase in in-hospital mortality (25.6% vs. 19.4%, p = 0.001). The DKA/HHS group also had higher incidences of acute kidney injury (39.4% vs. 18.9%, p < 0.001), sepsis (7.3% vs. 4.9%, p = 0.022), blood transfusion (11.3% vs. 8.2%) and a non-significant trend towards higher incidence of stroke (3.8% vs. 2.4%, p = 0.087). Also, DKA/HHS diagnosis was associated with lower rates of referral to coronary angiography (51.5% vs. 55.5%, p = 0.023), coronary stenting (26.1% vs. 34.8%, p < 0.001), or bypass grafting (6.2% vs. 8.7%, p = 0.033). Referral for invasive angiography was associated with lower odds of death during the hospitalization (*adjusted* OR 0.66, 95%CI 0.44-0.98, p = 0.039).

**Conclusions:**

Decompensated diabetes complicates ~ 1.5% of STEMI admissions in diabetic patients. It is associated with lower rates of referral for angiography and revascularization, and a negative differential impact on in-hospital morbidity and mortality and cost.

**Electronic supplementary material:**

The online version of this article (10.1186/s13098-018-0357-y) contains supplementary material, which is available to authorized users.

## Background

The impact of acute hyperglycemia on clinical outcomes of diabetic and non-diabetic patients admitted with ST-elevation myocardial infarction (STEMI) has been extensively studied. High admission blood glucose among STEMI patients is associated with higher short-term incidences of failed reperfusion, acute kidney injury, stent thrombosis, myocardial damage and death among [1–12]. In addition, hyperglycemia during STEMI hospitalizations in diabetics predicted left ventricular remodeling and survival during long-term follow-up [13–18]. However, large-scale outcomes data of STEMI patients with acutely decompensated diabetes manifesting as diabetic ketoacidosis (DKA) or hyperglycemic hyperosmolar state (HHS) are scarce [11]. We utilized a nationwide representative sample to assess the contemporary trends in the incidence and in-hospital morbidity and mortality and cost of decompensated diabetes (defined as DKA or HSS) among diabetic patients admitted with STEMI.

## Methods

### Study data

The National Inpatient Sample (NIS) was used to derive patient relevant information between January, 1st 2003 and December, 31st 2014. The NIS is the largest publicly available all-payer administrative claims-based database and contains information about patient discharges from approximately 1000 non-federal hospitals in 45 states. It contains clinical and resource utilization information on 5–8 million discharges annually, with safeguards to protect the privacy of individual patients, physicians, and hospitals. These data are stratified to represent approximately 20% of US inpatient hospitalizations across different hospital and geographic regions (random sample). National estimates of the entire US hospitalized population were calculated using the Agency for Healthcare Research and Quality sampling and weighting method.

### Study population

Patients > 18-year-old with a principle discharge diagnosis of ST-elevation myocardial infarction (International Classification of Diseases-Ninth Revision-Clinical Modification [ICD-9-CM] codes 410.×1 except 410.71) between 2003 and 2014 were identified in the NIS. Non-diabetic patients were then excluded yielding a cohort of diabetic patients admitted with STEMI. Those were then stratified into patients with or without decompensated diabetes. Similar to prior studies, we defined decompensated diabetes was defined as: diabetes with ketoacidosis (ICD-9-CM codes 250.10-250.13) or diabetes with hyperosmolaity (ICD-9-CM codes 250.20-250.23) [19, 20]. Myocardial infarction codes used in this study have a negative predictive value of 96.1%, and a positive predictive value of 95.9% and are similar to what has been reported in other studies [21, 22].

### Outcomes analysis and study endpoints

A comparative analysis was performed between diabetic patients admitted with STEMI with and without DKA/HSS before and after propensity score matching for the *primary end point* of in-hospital mortality, and for the s*econdary end points* of in-hospital complications, length of stay, post-discharge intermediate care utilization and cost.

To account for potential confounding factors and reduce the effect of selection bias, a propensity score-matching model was developed using logistic regression to derive two matched groups for the comparative outcomes analysis. Patients admitted with STEMI with or without DKA/HHS were entered into a nearest neighbor 1:1 variable ratio, parallel, balanced propensity-matching model using a caliper of 0.01 without replacement to ensure perfect matching. Variables included in the propensity match model are listed in Additional file 1: Table S1.

We anticipated that decompensated diabetes will have a differential impact on referral patterns for invasive angiography. We hence examined the impact of invasive angiography referral on in-hospital mortality among patients with decompensated diabetes using a multivariable logistical regression analysis model.

### Statistical analysis

Descriptive statistics presented as frequencies with percentages for categorical variables and as means with standard deviations for continuous variables. Baseline characteristics were compared using a Pearson Chi squared test and Fisher’s exact test for categorical variables and an independent-samples *t* test for continuous variables. Matched categorical variables were presented as frequencies with percentages and compared using McNamar’s test. Matched continuous variables were presented as means with standard deviations and compared using a paired-samples t-test. A type I error rate of < 0.05 was considered statistically significant. To assess monotonic trends of utilization and outcomes we employed the non-parametric Mann-Kendal trend method. All statistical analyses were performed using SPSS version 24 (IBM Corporation, Armonk, NY) and R, version 3.3.1.

## Results

**Fig. 1:**
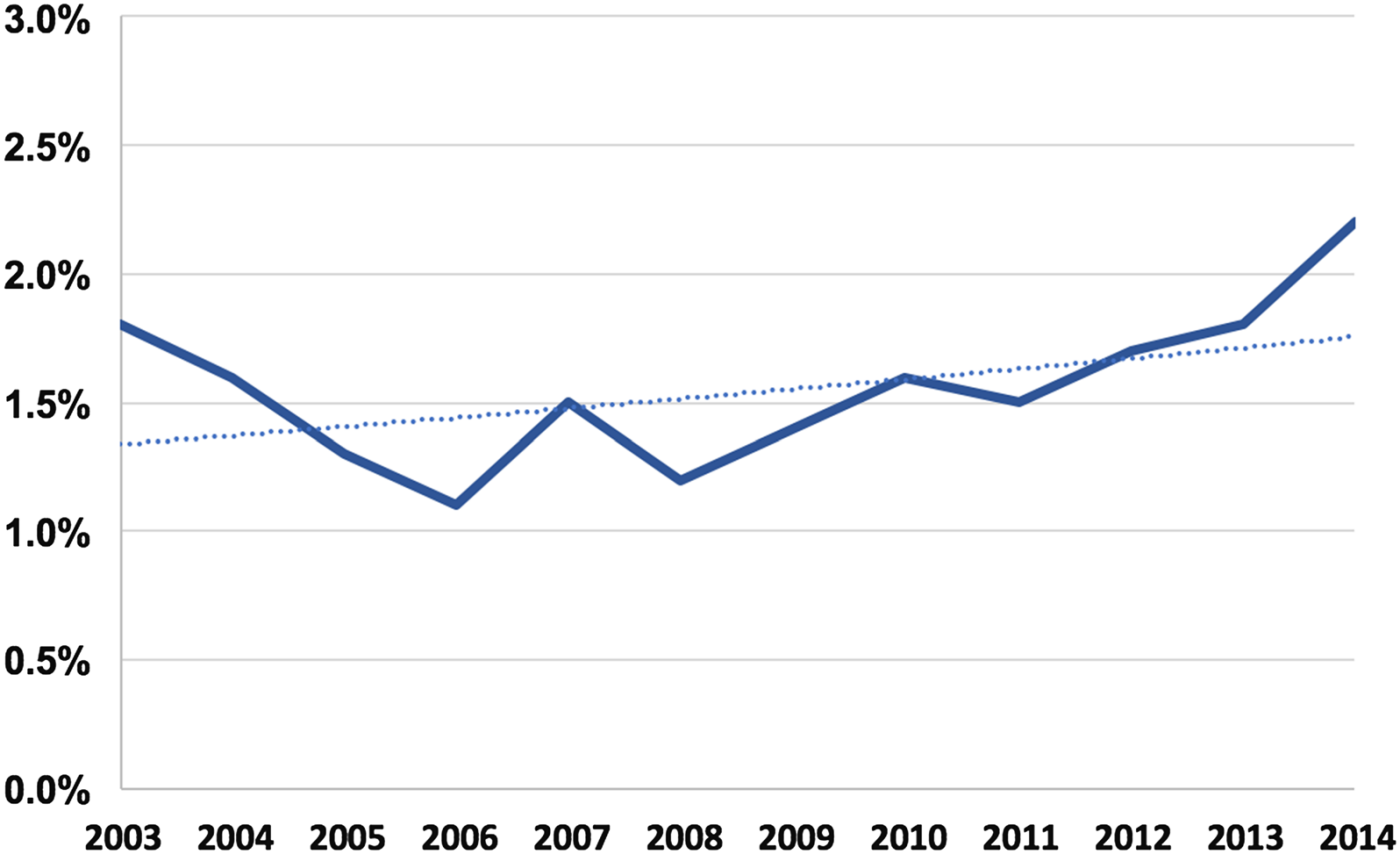
Trends in the incidence of decompensated diabetes among diabetic patients admitted with STEMI

A total of 73,722 diabetic patients (weighted national estimate = 362, 362) admitted with STEMI between January 1st, 2003 and December 31st, 2014 were included in the study. Of those, 72,591 patients (98.5%) had compensated diabetes and 1131 (1.5%) suffered DKA or HSS during the hospitalization with modest temporal increase in this incidence during the study period (Fig. 1). Patients with DKA/HHS were younger (63 ± 15 vs. 67 ± 15 years, p < 0.001) and a distinctive risk profile compared with diabetic patients without DKA/HHS, characterized with lower incidences of hypertension, hyperlipidemia, smoking, chronic obstructive lung disease, known coronary artery disease, and prior atrial fibrillation (Table 1).

**Table 1:** Baseline characteristics of the study’s population

Baseline characteristics	Compensated diabetes N = 72,591 NE = 356,810	Decompensated diabetes N = 1113 NE = 5553	*P* value
Age-mean (SD), years	64 ± 14	63 ± 15	< 0.001
Female	41.0%	47.3%	< 0.001
Race			0.752
Caucasian	70.5%	70.1%	
African American	10.5%	11.5%	
Hispanic	11.1%	11.2%	
Dyslipidemia	52.3%	32.4%	< 0.001
Hypertension	70.8%	51.7%	< 0.001
Prior sternotomy	7.3%	6.5%	0.361
Chronic lung disease	18.2%	13.7%	< 0.001
Atrial fibrillation/flutter	15.5%	12.0%	0.001
Anemia	16.1%	19.4%	0.003
Coagulopathy	4.0%	7.5%	< 0.001
Conduction abnormality	5.8%	7.2%	0.05
Congestive heart failure	0.7%	4.7%	< 0.001
Cardiogenic shock	9.3%	23.9%	< 0.001
Drug abuse	1.4%	3.3%	< 0.001
Smoking	26.6%	20.6%	< 0.001
Vascular disease	10.4%	8.8%	0.077
Coronary artery disease	64.6%	48.4%	< 0.001
Prior stroke	3.3%	2.6%	0.182
Chronic renal failure	17.4%	21.2%	0.001
Liver disease	1.1%	2.0%	0.005
Teaching hospital	43.2%	44.8%	0.285
Rural hospital location	15.1%	12.5%	0.016
Primary payer-no (%)			< 0.001
Medicare/medicaid	65.1%	62.0%	
Private insurance	25.3%	24.8%	
Self-pay	6.0%	9.8%	
No charge/other	3.7%	3.4%	

*SD* standard deviation

**Fig. 2:**
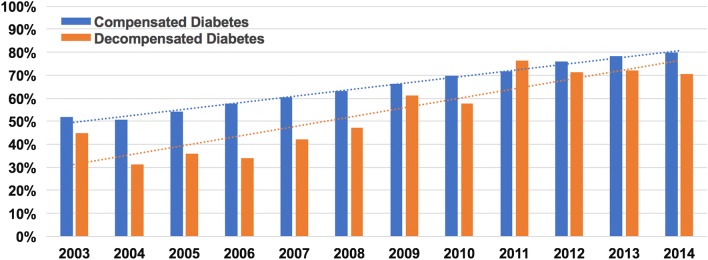
Trends in percutaneous coronary revascularization among diabetic patients admitted with STEMI

Patients with decompensated diabetes had higher incidences of cardiogenic shock (23.9% vs. 9.3%), and cardiac arrest (11.8% vs. 5.6%), and were less likely to undergo coronary angiography (51.4% vs. 61.1%), percutaneous coronary intervention (PCI) (25.8% vs. 40%), or coronary bypass grafting (6.2% vs. 9.2%) (P < 0.001 for all) (Tables 1, 2). However, referral to coronary angiography and PCI increased overtime (Fig. 2).

**Table 2:** Management patterns in the unmatched cohorts

Management	Compensated diabetes N = 72,591 NE = 356,810 (%)	Decompensated diabetes N = 1113 NE = 5553 (%)	*P * value
Coronary angiography	62.9	52.3	< 0.001
Coronary intervention	43.2	29.6	< 0.001
IV thrombolysis	1.8	1.3	0.218
Underwent PTCA	3.2	3.7	0.308
Underwent PCI	40.0	25.8	< 0.001
BMS	27.4	15.4	< 0.001
DES	13.4	10.8	< 0.001

*PTCA* percutaneous transluminal coronary angiography, *IV* intravenous, *PCI* percutaneous coronary intervention, *BMS* bare metal stent, *DES* drug eluting stent

### Outcomes of the unmatched cohorts

Compared with patients with decompensated diabetes, those with DKA/HHS had over twofold higher in-hospital mortality (26.4% vs. 11.9%, p < 0.001) and higher incidences of acute kidney injury (40% vs. 13.3%), stroke (3.7% vs. 1.6%), sepsis (7.5% vs. 1.9%), blood transfusion (11.3% vs. 8.2%) and mechanical ventilation (8.6% vs. 2.9%), (p < 0.001 for all). They also had longer hospitalizations (7 ± 8 vs. 5 ± 6 days, p < 0.001), higher hospital charges (87,382 ± 132,119$ vs. 64,396 ± 81,185$, p < 0.001), and were less likely to be discharged directly to home (58.7% vs. 68.4%, p < 0.001) (Table 3).

**Table 3:** In-hospital outcomes of STEMI patients with and without DKA/HHS

Clinical outcome	Unmatched cohorts			Matched cohorts		
	Compensated diabetes N = 72,591 NE = 356,810 (%)	Decompensated diabetes N = 1113 NE = 5553 (%)	*P* *value*	Compensated diabetes N = 1103 NE = 5410 (%)	Decompensated diabetes N = 1103 NE = 5410 (%)	*P * value
Death	11.9	26.4	< 0.001	19.4	25.6	0.001
Blood transfusion	8.2	11.3	< 0.001	11.7	11.1	0.689
Acute kidney injury	13.3	40.0	< 0.001	18.9	39.4	< 0.001
New dialysis	0.4	0.5	0.521	0.5	0.5	0.99
Stroke	1.6	3.7	< 0.001	2.4	3.8	0.087
UTI	7.4	13.1	< 0.001	8.3	13.1	0.001
Sepsis	1.9	7.5	< 0.001	4.9	7.3	0.022
Acquired pneumonia	6.6	12.0	< 0.001	7.5	12.1	< 0.001
Mechanical ventilation	2.9	8.6	< 0.001	7.1	8.0	0.456
Tracheostomy	0.6	1.5	<0.001	2.6	1.0	0.006
Discharged home	60.3	43.3	< 0.001	51.0	44.1	0.01
Discharged to IC	26.9	29.1		28.3	29.2	
LOS (mean ± SD)	5 ± 6	7 ± 8	< 0.001	6 ± 7	7 ± 7	0.02
Total charges ($)	64,396 ± 81,185	87,382 ± 132,119	< 0.001	76,983 ± 109,872	84,527 ± 125,828	0.133

*UTI* urinary tract infection, *IC* intermediate care facility, *SD* standard deviation, *LOS* length of stay

### Outcomes of the matched cohorts

Following propensity-score matching, baseline characteristics between the two sets of STEMI patients with and without DKA/HHS became well matched (Additional file 1: Table 2 and Figure S1). The presence of DKA/HHS remained associated with significantly lower rates of referral to coronary angiography (52.2% vs. 57.1%, p = 0.023), and PCI (29.5% vs. 38.4%, p < 0.001) (Additional file 1: Table S3). In these propensity-matched cohorts, DKA/HHS remained associated with a significant 32% increase in in-hospital mortality (25.6% vs. 19.4%, p = 0.001). The DKA/HHS group also had significantly higher incidences of acute kidney injury (39.4% vs. 18.9%, p < 0.001), sepsis (7.3% vs. 4.9%, p = 0.022), and a non-significant trend towards higher stroke (3.8% vs. 2.4%, p = 0.087). Similar to the unmatched cohorts, they also had longer hospitalizations, higher hospital charges and were less likely to be discharged directly to home (Table 3).

Given that patients in the decompensated diabetes group were less likely to be referred for invasive angiography and they also had higher in-hospital mortality, the impact of invasive angiography referral on in-hospital mortality among these patients was examined using a multivariable logistical regression analysis model. This model adjusted for demographics, baseline clinical risk factors, insurance status and hospital characteristic, and showed that referral for invasive angiography was associated with lower odds of death during the hospitalization (*adjusted* OR 0.66, 95%CI 0.44–0.98, p = 0.039). Variables included in this regression models are the same variables used for propensity score matching outlined in Additional file 1: Table S1.

## Discussion

The main findings of the present investigation are: (1) acute diabetes decompensation occur in 1.5% of diabetics admitted with STEMI, (2) those patients have less prevalence of previously diagnosed coronary artery disease, but higher incidences of cardiogenic shock and cardiac arrest, (3) patients with decompensated diabetes were less likely to undergo coronary angiography and revascularization compared with patients with compensated diabetes, (4) referral to angiography was independently associated with lower risk-adjusted odds of in-hospital death in patients with decompensated diabetes, and (5) acute diabetes decompensation was associated with 32% higher in-hospital mortality, higher incidences of certain key morbidities, more resource utilization, and higher cost.

Prior studies have shown that the hyperglycemia during STEMI admissions is not uncommon in both diabetics and non-diabetics, and hyperglycemia in this setting is associated with worse short and long-term outcomes [1–18]. Nonetheless, to our knowledge, the incidence of full-scale uncontrolled hyperglycemia (decompensated diabetes) manifesting as DKA or HHS and its impact on clinical outcomes among STEMI patients have not been previously investigated.

Our analysis reveals that the incidence of DKA or HHS among diabetic patients admitted with STEMI is low (1.5%) but is associated with lower odds of undergoing revascularization, and higher morbidity and mortality and cost. Albeit intuitive, the findings of our study deserve more scrutiny:

1. The lower prevalence of known coronary artery disease and the worse initial presentation (evident by the higher incidence of cardiogenic shock and cardiac arrest) in the DKA/HHS population are absorbing. Although speculative, these patients may have constituted a different cohort of patients at baseline that is characterized with worse chronic glycemic control, attenuated symptoms due to diabetic neuropathy, delayed presentations, and possibly larger infarcts [23, 24].
2. The interrelation between DKA/HHS and worse outcomes in STEMI patients is rather complex and may be difficult to unravel from cohort analyses like the present one. Several studies have shown than uncontrolled hyperglycemia is associated with high plasma catecholamine levels, oxidative stress, and endothelial and microvascular dysfunction possibly leading to higher incidence of no reflow, larger infarcts and hence worse STEMI outcomes [1, 25, 26]. Vice versa, the occurrence of DKA/HHS during a STEMI admission may be related to worse STEMI profile in these patients (cardiogenic shock or cardiac arrest). These patients may experience more intense hemodynamic and hormonal derangements and hence may develop DKA or HHS as a result for their ‘higher-risk’ STEMI. Further studies are needed to delineate the impact of variable degrees of hyperglycemia on STEMI outcomes and to identify the associated underlying mechanisms.
3. Patients with DKA/HHS were less likely to be referred to angiography, although this has improved with time. Reasons for this ‘less invasive’ practice are not known and can not be ascertained in our study. Nonetheless, referral for angiography was independently associated with improved in hospital survival suggesting a potential opportunity for improvement in this cohort.
4. The excess associated mortality of DKA/HHS was significantly attenuated after rigorous propensity score matching: 26.4% vs. 11.9%, p < 0.001 before matching, and 25.6% vs. 19.4%, p = 0.001 after matching. This suggests that baseline and hospital characteristics played a key role in the variance of outcomes between the two groups.

## Limitations

Our study has a number of limitations. (1) The NIS is an administrative database that gathers data for billing purposes and can be limited by erroneous coding. Nevertheless, the Healthcare Cost and Utilization Project’s quality control minimizes the discrepancies related to diagnosis coding. Also, both the procedures and the hard clinical endpoints reported in our study are hard to miscode. (2) Angiographic data, and information on peri-procedural hemodynamics, medications use, are not available in the NIS. (3) Similarly, granular data on blood glucose levels, and insulin use as well as the timing of the DKA/HHS diagnoses are not captured in NIS. (5) Pre-admission diabetic control and anti-diabetic medications are not included in this dataset. Several studies have suggested a possible impact of Metformin and other anti-diabetics on infarct size and outcomes in diabetic patients admitted with STEMI [27–29]. This potential effect can not be assessed with the current study design. (4) Significant differences in baseline risk profiles were noted between the two groups. The impact of unmeasured confounders on the comparative analysis cannot be ruled out. Nonetheless, we believe that our rigorous propensity score matching should minimize that risk.

## Conclusions

Decompensated diabetes complicates ~ 1.5% of STEMI admissions in diabetic patients. It is associated with lower rates of referral for angiography and revascularization, and a negative differential impact on in-hospital morbidity and mortality and cost. Further studies are needed to identify preventative and management strategies to mitigate the excess morbidity and mortality in these patients.

## Additional file

**Additional file 1: Table S1.** Variables used for propensity score matching. **Table S2.** Baseline characteristics of the propensity matched groups. **Table S3.** Management patterns in the propensity matched cohorts. **Figure S1.** Standardized mean differences before and after propensity score matching.
