# Supplementary material for: Impact of acute diabetes decompensation on outcomes of diabetic patients admitted with ST-elevation myocardial infarction
Source: Diabetol Metab Syndr. 2018 Jul 17;10:57. doi: 10.1186/s13098-018-0357-y (PMC6050700; doi:10.1186/s13098-018-0357-y)
Supplement: Supplementary file 1 — Additional file 1: Table S1. Variables used for propensity score matching. Table S2. Baseline characteristics of the propensity matched groups. Table S3. Management patterns in the propensity matched cohorts. Figure S1. Standardized mean differences before and after propensity score matching. [file 13098_2018_357_MOESM1_ESM.docx]

|  |  |
| --- | --- |
| Age  Race  Gender  Hypertension  Prior Sternotomy  Chronic Pulmonary disease  Atrial fibrillation\flutter  Congestive heart failure  Chronic kidney disease  Chronic Anticoagulation  Coagulopathy  Conduction abnormality  Drug Abuse  Smoking  Peripheral vascular disease  Pulmonary hypertension  Obesity  Coronary artery disease  Blood loss anemia | Iron deficiency anemia Neurological disorders  Hypothyroidism  Liver disease  Deep Venous Thrombosis  Metastatic disease  Solid tumors without metastasis  Collagen vascular disease  Weight loss  Alcoholism  Depression  Drug abuse  Psychosis  Hospital teaching status  Hospital Bed Size  Rural Location  Region of the Hospital  Primary Payer  Median Household Income |

**Table S1: Variables Used for Propensity Score Matching**

| **Baseline**  **Characteristics** | **Decompensated Diabetes**  N=1,103  NE= 5,410 | **Compensated Diabetes**  N=1,103  NE= 5,410 | ***P value*** |
| --- | --- | --- | --- |
|  | N=1,103 | N=1,103 |  |
| **Age** | 63±14 | 63±15 | 0.96 |
| **Race- no. (%)** |  | | 0.057 |
| **Caucasian** | 70.7% | 69.4% |  |
| **African American** | 11.4% | 11.2% |  |
| **Hispanic** | 11.2% | 11.8% |  |
| **Dyslipidemia** | 33.3% | 33.1% | 0.961 |
| **Hypertension** | 52.1% | 52.8% | 0.781 |
| **Prior Sternotomy** | 5.8% | 6.5% | 0.53 |
| **Chronic Lung Disease** | 14.0% | 13.9% | 0.99 |
| **Atrial Fibrillation/Flutter** | 12.0% | 12.3% | 0.844 |
| **Anemia** | 18.5% | 19.5% | 0.595 |
| **Coagulopathy** | 6.9% | 7.1% | 0.934 |
| **Conduction Abnormality** | 7.2% | 7.0% | 0.933 |
| **Congestive Heart Failure** | 4.1% | 3.7% | 0.743 |
| **Cardiogenic Shock** | 22.7% | 22.7% | 0.99 |
| **Drug Abuse** | 2.6% | 3.1% | 0.609 |
| **Smoking** | 20.0% | 20.9% | 0.67 |
| **PVD** | 10.0% | 8.9% | 0.427 |
| **Coronary Artery Disease** | 48.0% | 49.3% | 0.522 |
| **Prior CVA** | 2.4% | 2.6% | 0.779 |
| **Chronic Renal Failure** | 22.0% | 21.2% | 0.678 |
| **Liver Disease** | 97.3% | 98.0% | 0.322 |
| **Teaching Hospital** | 45.6% | 44.3% | 0.583 |
| **Rural hospital location** | 12.3% | 12.5% | 0.951 |
| **Primary Payer- no (%)** |  | | 0.189 |
| **Medicare / Medicaid** | 64.1% | 62.6% |  |
| **Private Insurance** | 23.8% | 24.6% |  |
| **Self-pay** | 9.0% | 9.4% |  |
| **No charge/Other** | 3.1% | 3.4% |  |

**Table S2 Baseline Characteristics of the Propensity Matched Groups**

SD; standard deviation, Y; year

| **Management** | **Compensated Diabetes**  N=1,103  NE= 5,410 | **Decompensated Diabetes**  N=1,103  NE= 5,410 | ***P value*** |
| --- | --- | --- | --- |
| **Coronary Angiography** | 57.1% | 52.2% | 0.023 |
| **Coronary Intervention** | 38.4% | 29.5% | <0.001 |
| **- IV Thrombolytic** | 1.8% | 1.3% | 0.391 |
| **- Underwent PTCA** | 3.6% | 3.3% | 0.727 |
| **- Underwent PCI** | 34.8% | 26.1% | <0.001 |
| ***BMS** | 13.5% | 10.9% | 0.069 |
| ***DES** | 21.7% | 15.6% | <0.001 |

**Table S3 Management Patterns in the Propensity Matched Cohorts**

PTCA; percutaneous transluminal coronary angiography, IV; intravenous, PCI, percutaneous coronary intervention, BMS; bare metal stent, DES; drug eluting stent.


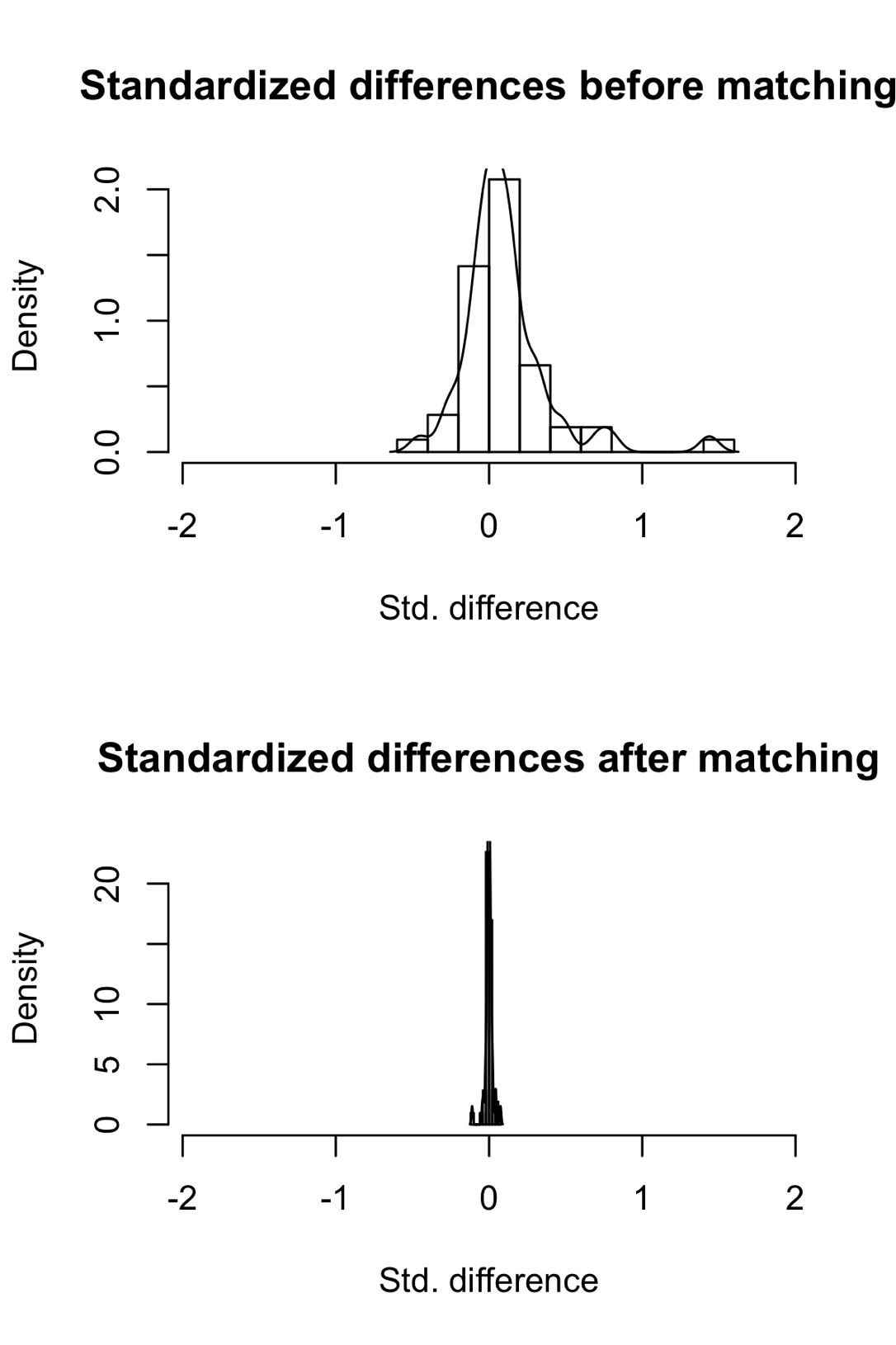


**Figure S1:**

**Standardized Mean Differences Before and After Propensity Score Matching**
